# Molecular pathways underlying amyloid precursor protein–mediated regulation of adult-born neurons

**DOI:** 10.4103/NRR.NRR-D-25-00175

**Published:** 2025-11-25

**Authors:** Haidong Hu, Huidong Li, Yu Chen, Jianwen Zhou, Jian Chen, Qihong Tang, Xiaoshan Chen, Jinxiang Jiang, Mengyao Sun, Dongjing Jia, Wenyuan Xie, Cheng Long, Li Yang

**Affiliations:** 1 School of Life Sciences, Guangzhou University, Guangzhou, Guangdong Province, China; 2 School of Life Sciences, South China Normal University, Guangzhou, Guangdong Province, China

**Keywords:** adult-born granule cells, Alzheimer’s disease, amyloid precursor protein, apoptosis, fear conditioning, hippocampus, parvalbumin, potassium-chloride-cotransporter 2, whole-cell patch-clamp

## Abstract

Cleavage of amyloid precursor protein (APP) produces toxic amyloid-beta peptides, which play a critical role in the pathogenesis of Alzheimer’s disease. Neuronal loss is a key feature of Alzheimer’s disease. Despite the importance of APP in the proliferation of neural progenitors and the survival of adult-born granule cells in the dentate gyrus, little is known about the effect of APP deficiency on neuronal electrophysiological activities and the survival of newly born neurons. Utilizing whole-cell patch-clamp recording in combination with retroviral labeling and immunofluorescent staining in Alzheimer’s disease model mice with *App* knockout (*App*^–/–^), we show that APP deficiency increased the number of adult-born granule cells at 4 weeks post-injection, but did not affect their intrinsic excitability or miniature current activity. In contrast, at 10 weeks post-injection, adult-born granule cells showed increased abundance and intrinsic excitability that were associated with abnormal dendritic morphology, increased miniature excitatory- and inhibitory-synaptic transmission, and decreased potassium-chloride-cotransporter 2 expression. Compared with adult-born granule cells at 10 week post-injection, mature granule cells exhibited decreased intrinsic excitability and potassium-chloride-cotransporter 2 expression alongside increased apoptosis in *App*^–/–^ mice. Additionally, although *App*^–/–^mice showed abnormal freezing behavior and elevated mature granule cell activation during contextual fear conditioning, adult-born granule cells were not recruited in either *App*^–/–^ or wild-type control mice. Taken together, these findings suggest that APP is required for adult-born granule cell maturation and that APP deficiency induces excitotoxicity in adult-born granule cells at 10 weeks post-injection, promoting subsequent apoptosis of mature granule cells.

## Graphical abstract

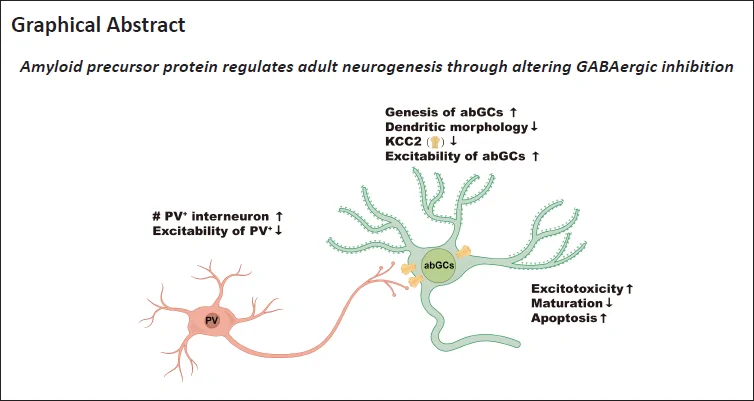

## Introduction

Alzheimer’s disease (AD), a neurodegenerative condition, is characterized by deposition of amyloid-beta (Aβ) plaques, neurofibrillary tangles (NFTs), and impairments in learning, memory, and executive function, including instrumental performance and activities of daily living (Weller and Budson, 2018; Breijyeh and Karaman, 2020; Se Thoe et al., 2021). Its two key pathological features, deposition of Aβ and hyperphosphorylation of tau protein, result in the formation of NFTs (Monteiro et al., 2023; Rajendran and Krishnan, 2024). Aβ plaques consist of abnormal aggregates of Aβ peptides comprising 40 or 42 amino acids (Aβ_1–40_ and Aβ_1–42_), which represent cleavage products of amyloid precursor protein (APP) (Brunholz et al., 2012; Ono and Watanabe-Nakayama, 2021; Cho et al., 2022). While considerable attention has been given to the pathogenic role of Aβ and the development of targeted anti-Aβ drugs (Zhang et al., 2021, 2023; Vitek et al., 2023), the possible contribution of APP loss-of-function to AD pathogenesis remains unclear. As an extensively processed type I transmembrane protein, APP and its metabolites have been shown to affect neuronal functions, including membrane ion channel activity (Yang et al., 2009; Chen et al., 2017), synaptic transmission and neural oscillations (Dawson et al., 1999; Huo et al., 2017), and cognitive performance (Tambini et al., 2020; Gabriele et al., 2022). APP plays multiple roles in various brain regions, where it is highly expressed in both excitatory neurons and gamma-aminobutyric acid (GABA) interneurons (Cho et al., 2022; Wang et al., 2014). We previously showed that APP deficiency in mice impairs the expression and function of hippocampal voltage-gated L-type calcium channel subtype 1.2 (Cav1.2) (Yang et al., 2009) and negatively affects GABAergic synaptic transmission by regulating potassium-chloride-cotransporter 2 (KCC2) protein (Chen et al., 2017). APP plays a key role in modulating neural oscillations in the medial prefrontal cortex (Huo et al., 2017) and interacts with the high-affinity choline transporter to affect cholinergic synaptic function (Wang et al., 2007; Cai et al., 2016). However, its precise role within specific neuronal subtypes remains largely unclear.

The hippocampus, which is involved in processing spatial and environment-related fear memory, is one of the brain regions most critically affected by AD (Rao et al., 2022; Danieli et al., 2023; Pahlavani, 2023). Neural stem cells generate new neurons throughout life in specific areas of the mammalian brain, including the dentate gyrus (DG) of the hippocampus (Frechou et al., 2024), where adult-born granule cells (abGCs) are generated in both rodents (Kim et al., 2021; Li et al., 2022; Shen et al., 2022) and humans (Sorrells et al., 2018; Moreno-Jimenez et al., 2021; Gage, 2025). The DG is highly vulnerable to age-related pathology (Babcock et al., 2021; Culig et al., 2022; Wu et al., 2025). Adult neurogenesis in the DG is regulated by a number of environmental and cell-intrinsic factors in response to physiological and pathological changes (Amanollahi et al., 2023; Fang et al., 2023; Gage, 2025). Despite these insights, little is known about how APP regulates abGC genesis and maturation in the adult brain.

Neuron loss and diminished adult neurogenesis, both key characteristics of AD, result in learning and memory deficits (Wirths and Zampar, 2020; Choi and Tanzi, 2023; Grabrucker et al., 2023; Salta et al., 2023). Given the crucial role of APP in AD pathogenesis and the DG as a source of adult neurogenesis, understanding how APP influences DG physiology, particularly in abGCs, is essential. A previous study using immunohistochemical labeling of mature neurons and *in situ* hybridization-mediated labeling of immature neurons in a transgenic AD mouse model overexpressing human APP showed that the abGCs did not fully mature, despite increased neuroproliferation (Li et al., 2008). Although *App* knockout (*App*^–/–^) mice exhibit increased neuroproliferation and reduced abGC survival in the DG (Wang et al., 2014), it remains unclear how the APP deficiency impairs the electrical and molecular properties of abGCs, and thus their survival. Also unresolved is the role of altered GABA signaling and its molecular mediators in regulating abGC electrophysiology in the DG of *App*^−/−^ mice.

The DG receives input from the entorhinal cortex and transduces signals to the hippocampal subfields cornu ammonis (CA)3 and CA1 (Meier et al., 2020), contributing critically to the initiation, retrieval, and extinction of fear memory (Zhang et al., 2021; Gong et al., 2022; Cui et al., 2024). Aging-associated decline in fear memory worsens in patients and rodents under AD conditions (Dulka et al., 2020; Maturana et al., 2023). Impaired learning and memory, such as deficits in classical fear conditioning, is a key feature of AD (Hamann et al., 2002; Hoefer et al., 2008), and abnormal extinction of fear memories occurs in early-stage AD (Nasrouei et al., 2020). Defective fear conditioning has been proposed as an early predictive biomarker of AD (Hoefer et al., 2008; Nasrouei et al., 2020). APP and its cleavage products play crucial roles in fear memory. Deficiency of Aη, a recently identified APP cleavage product, alters N-methyl-D-aspartate receptor-mediated synaptic transmission, prevents the induction of long-term depression, and impairs contextual fear memory (Wang et al., 2009; Lelos and Good, 2014; Dunot et al., 2024). Similarly defective long-term potentiation and fear memory caused by extracellular Aβ and tau oligomers are APP-dependent (Puzzo et al., 2011), and the effects of soluble oligomeric forms of human Aβ and NFTs on contextual fear memory can be abolished by deleting *App* in mice (Puzzo et al., 2017). However, whether abGCs are differently activated by contextual fear conditioning (CFC) in *App*^−/−^ *versus* wild-type (WT) mice remains unknown.

The *App*^−/−^ mouse model and analyses of related genes and proteins have been used to obtain insights into the critical role of APP in the brain (Beglopoulos and Shen, 2004). In this study, we used retroviral labeling to track abGCs in the DG (Denoth-Lippuner and Jessberger, 2021; Wang et al., 2014) of WT control and *App*^−/−^ mice, combined with biochemical assays and whole-cell patch-clamp recordings in brain slices, to examine the morphological, biochemical, and electrical properties of abGCs at 4 and 10 weeks post-injection (wpi), as well as in mature granule cells (mGCs). While APP deficiency did not affect key electrophysiological properties of 4-wpi abGCs, we observed altered excitability and synaptic activity in 10-wpi abGCs and mGCs, defined here as neurons born embryonically or postnatally prior to retroviral labeling and thus not tagged by green fluorescent protein (GFP). To investigate the mechanisms underlying these alterations, we combined immunofluorescent staining for KCC2, c-Fos, and parvalbumin-positive (PV^+^) interneurons with terminal deoxynucleotidyl transferase dUTP nick-end labeling (TUNEL) assays for cell death, alongside morphological, behavioral, and electrophysiological analyses. These approaches reveal how APP regulates the genesis, maturation, and functional integration of abGCs in the DG.

## Methods

### Animals and viruses

*App*^+/−^ mice were purchased from the Model Animal Research Center of Nanjing University (Nanjing, China; License No. SYXK (Su) 2024-0001). *App*^−/−^ and WT mice were produced by intercrossing *App*^+/−^ males and females. To label PV^+^ interneurons for whole-cell patch-clamp recording, we crossed PV-Cre^+^ mice (National Institute of Biological Sciences, Beijing, China) with *App*^+/−^ mice to produce PV-Cre^+^;*App*^+/+^ and PV-Cre^+^;*App*^−/−^ mice. Both male and female 1- to 3-month-old mice were used for the experiments. Microinjection of an adeno-associated virus (AAV) carrying a double-floxed inverted open reading frame (DIO) of human synapsin promoter (hSyn)-driven enhanced green fluorescent protein (EGFP), AAV-hSyn-DIO-EGFP (Feng et al., 2021), into the DG of PV^Cre+^ mice gave rise to mice with EGFP-expressing PV^+^ interneurons. A retroviral vector (RV) encoding EF1A promoter-driven EGFP and a U6-driven short hairpin RNA (shRNA) cassette, pROV-U6-shRNA-EF1A(S)-EGFP, hereafter referred to as RV-GFP, was from Baylor College of Medicine (Houston, TX, USA). Animal treatment and experiments were approved by the Ethics Committee for Animal Research at Guangzhou University and South China Normal University, and followed the National Institutes of Health Guidelines for the Care and Use of Laboratory Animals. Efforts were made to minimize animal suffering during surgery and to reduce the number of animals used. All experimental animals were group-housed (maximum of five mice per cage) under standard laboratory conditions of temperature (24 ± 1°C) and humidity (55% ± 5%), with a 12-hour light–dark cycle, and had free access to food and water.

### Stereotaxic surgery and viral injection

Stereotaxic injection of RV-GFP or AAV-hSyn-DIO-EGFP into the DG of 6- to 7-week-old mice was performed following an established protocol (Jiang et al., 2023), and resulted in GFP labeling of newly generated granule cells (GCs) (van Praag et al., 2002; Zhao et al., 2006; Toni et al., 2008) or PV interneurons (Feng et al., 2021). Mice were anesthetized with isoflurane (RWD Life Science Co., Ltd., Shenzhen, Guangdong, China) at 5% for induction and maintained at 1.5%–2%, delivered via nose cone. Mice were head-fixed in a stereotaxic frame (RWD Life Science Co. Ltd.) and care was taken to properly secure the head. Rectal temperature was maintained at 37°C using a temperature controller (Harvard Apparatus, Holliston, MA, USA). The surgical area was disinfected with iodophor, and the skin was excised to expose the skull. The meningeal layer was removed to fully expose the anterior and posterior fontanels. Small, bilateral craniotomies were performed, followed by bilateral viral injections targeting the DG at the following coordinates relative to the bregma: anteroposterior, –1.9 mm; dorsoventral, –1.9 mm; mediolateral, ±1.5 mm. Coordinates were confirmed using the Allen Mouse Brain Atlas (http://atlas.brain-map.org) and cross-validated with The Mouse Brain in Stereotaxic Coordinates (Paxinos and Franklin, 2013). Each hemisphere received either 2 μL of RV at a rate of 120 nL/min via glass pipettes connected to a 5-μL Hamilton syringe (Hamilton Co., Reno, NV, USA), or 300 nL of AAV at 60 nL/min via a 1-μL Hamilton syringe. Following infusion, the pipettes were left in place for an additional 10 minutes to allow viral diffusion before being slowly withdrawn at a rate of 0.2 mm/s. After surgery, the wound was sutured, disinfected, and the animal was returned to its home cage upon awakening. Animals injected with RV remained in their home cages for 4 or 10 weeks to allow viral expression, while animals injected with AAV remained in their home cages for 3 weeks.

### Whole-cell patch-clamp recordings

Mice were anesthetized with an intraperitoneal (i.p.) injection of pentobarbital (80 mg/kg; Sigma-Aldrich, St. Louis, MO, USA) and then transcardially perfused with ice-cold and oxygenated N-methyl-D-glucamine-based modified artificial cerebrospinal fluid (aCSF) (Li et al., 2025) The brain was quickly removed, placed in ice-cold N-methyl-D-glucamine-based aCSF, and mounted on a specimen holder using adhesive. Coronal slices containing the DG (thickness: 350 μm) were prepared using a Leica VT1000S vibratome (Leica, Wetzlar, Germany) and maintained in ice-cold N-methyl-D-glucamine-based aCSF. For recording, the slices were transferred to a holding chamber containing HEPES-based aCSF, as previously described (Feng et al., 2021), and incubated for at least 1 hour at room temperature before being transferred to a recording chamber and continuously perfused with oxygenated aCSF (Feng et al., 2021) at a rate of 2 mL/min. All solutions were maintained at a pH of 7.2–7.4 and were bubbled with 95% O_2_/5% CO_2_ throughout the experiment. All recordings were performed using 5–6 MΩ pipettes, a MultiClamp 700B amplifier, and a 1440A digitizer (Molecular Devices, San Jose, CA, USA). Internal solutions for measuring action potentials were prepared as previously described (Li et al., 2025). Miniature excitatory and inhibitory postsynaptic currents (mEPSCs and mIPSCs, respectively) were recorded using an established solution (Li et al., 2025).

All recordings were performed at room temperature. For AP recordings, neurons were held in current-clamp mode and stimulated with a series of current pulses (–70 to +260 pA in 10-pA increments). Membrane input resistance was calculated in response to a series of hyperpolarizing pulses. For rheobase testing, a ramped current stimulus (0 to +260 pA within 500 ms) was applied to GCs in the DG. Only recordings with stable series resistances (< 20% variation) and normal membrane potentials (more negative than –60 mV) were analyzed. For mEPSC and mIPSC recordings, neurons were clamped at –60 mV or 0 mV, respectively, in the presence of 300 nM tetrodotoxin in recording solution. Action potentials were measured in the absence of any neurotransmitter blockers. To isolate mEPSCs, bicuculline (10 μM) was added; for mIPSCs, 6-cyano-7-nitroquinoxaline-2,3-dione (CNQX; 20 μM) and D-2-amino-5-phosphonovaleric acid (APV; 50 μM) were added. Recordings of mGCs were conducted in 2- to 2.5-month-old mice. Data were acquired with a 2-kHz low-pass filter using pClamp 10 and analyzed offline using Clampfit 10.6 (Molecular Devices).

### Immunohistochemistry

Immunofluorescent staining was conducted following a published protocol (Jiang et al., 2023). Mice were anesthetized with i.p. urethane (2 g/kg; Sigma-Aldrich), perfused with ice-cold phosphate-buffered saline (PBS), followed by 4% paraformaldehyde in PBS. The brain was postfixed in 4% paraformaldehyde at 4°C for 12 hours, then cryoprotected by immersion in 15% sucrose overnight, followed by 30% sucrose until the tissue no longer floated. It was then cut into 30-μm-thick sections using a Leica CM30505 freezing microtome. The dorsal portion of DG-containing sections was permeabilized with 0.3% Triton X-100 and 5% donkey serum in PBS for 2 hours. The sections were then incubated with one of the following antibodies at 4°C overnight: rabbit polyclonal anti-PV (1:1000, Abcam, Cambridge, UK, Cat# ab11427, RRID: AB_298032), anti-KCC2 (1:100, Merck KGaA, Darmstadt, Germany, Cat# 07-432, RRID: AB_310611), anti-c-Fos (1:800, Abcam, Cat# ab190289, RRID: AB_2737414), anti-tropomyosin-related kinase receptor type B (TrkB; 1:1000, Merck Millipore, Darmstadt, Germany, Cat# abN1381, RRID: AB_2721199), anti-GABA_A_Rα1 (1:1000, Merck Millipore, Cat# 06-868, RRID: AB_310272), anti-brain-derived neurotrophic factor (BDNF; 1:1000, Abcam, Cat# ab205067), anti-AMPAR1 (1:1000, Abcam, Cat# ab31232, RRID: AB_2113447), and anti-GABA_A_Rα5 (1:1000, Abcam, Cat# ab242001). After three washes with PBS, the sections were incubated with goat anti-rabbit Alexa Fluor 594 secondary antibody (1:1000, Thermo Fisher Scientific, Cat# A-11012, RRID: AB_2534079) for 1.5 hours at room temperature, followed by three 10-minute washes in PBS. Finally, the sections were incubated with 4,6-diamidino-2-phenylindole (DAPI; Beyotime, Jiangsu, China, Cat# P0131) to label nuclei, and coverslipped with anti-fade mounting medium. Images were captured using a Nikon Eclipse Ni-E fluorescence microscope (Nikon, Tokyo, Japan) and processed by ImageJ software version 1.8.0 (National Institutes of Health, Bethesda, MD, USA).

### TUNEL assay

Intrahippocampal injection of RV-GFP was performed to label abGCs in WT and *App*^−/−^ mice. At 10 wpi, frozen brain sections from these mice were stained using a TUNEL assay kit (Roche, Basel, Switzerland; Cat# 12156792910) following the manufacturer’s instructions. Briefly, sections were fixed with 4% paraformaldehyde for 20 minutes at room temperature, washed with PBS for 30 minutes, and incubated in permeabilization solution (0.1% Triton X-100 and 0.1% sodium citrate in PBS) for 2 minutes on ice. After rinsing twice with PBS, sections were incubated in TUNEL reaction mixture in a humidified atmosphere for 1 hour at 37°C in the dark. Sections were then thrice washed with PBS (5 minutes each) and coverslipped using antifade mounting medium containing DAPI. Images were acquired in 2-μm z-steps using a Zeiss LSM-800 confocal microscope (Zeiss, Jena, Germany). Image processing and analysis were performed using ZEN software (Zeiss).

### Dendritic morphology

Images of RV-GFP-labeled abGCs in the DG of WT and *App*^−/−^ mice at 10 wpi were obtained in 0.2-μm z-steps using a Zeiss LSM-800 confocal microscope (40× oil immersion). Images were then processed and quantified by blinded assessors using ZEN and ImageJ software. The abGCs were traced using the NeuronJ plugin of ImageJ and analyzed for total dendritic length and surface area. Sholl analysis performed using concentric circles spaced at 10-μm intervals was employed to quantify dendrite branching. For dendritic spine quantification, 5–7 abGCs per mouse and 2–4 tertiary apical dendrites of comparable diameter (> 20 μm) per abGC were analyzed. Spine density was calculated as the number of spines per unit dendritic length.

### Behavioral test

Fear conditioning was performed using the Panlab StartFear Combined system (Harvard Apparatus), which is based on weight sensing, following a published procedure (Matos et al., 2019; Li et al., 2021). Briefly, for 2 days prior to the behavioral experiment, mice were moved to the behavioral room once daily for 3 hours per day to allow for adaptation. During the conditioning stage, mice were allowed to freely explore the situational fear box for 180 seconds before receiving three plantar electric shocks (0.7 mA, 2 seconds each) spaced 58 seconds apart. After the final electric shock, mice were left in the fear box for 1 minute before being returned to the home cage. Testing was conducted the next day, when mice were returned to the same contextually conditioned fear box for 5 minutes, without shock. Freezing behavior during this 5-minute period was quantified as a measure of contextual fear. The fear box was wiped with 75% alcohol after each session to remove any residual odor. Freezing frequency (%) at each behavioral stage was recorded and analyzed using PACKWIN software (Harvard Apparatus). Brains were perfused for 90 minutes post testing for subsequent c-Fos immunostaining.

### Statistical analysis

Statistical analysis was performed using SPSS software (version 25.0; IBM, Armonk, NY, USA), and data were plotted using Origin 2018 (OriginLab, Northampton, MA, USA). Data were analyzed using a two-tailed unpaired Student’s *t*-test or two-way repeated-measures analysis of variance with Tukey’s *post hoc* test. Data are presented as mean ± standard error of the mean (SEM). Statistical significance was set as a *P* value < 0.05.

## Results

### Action potentials and membrane properties of adult-born granule cells at 4 and 10 weeks post-injection

**Figure 1:**
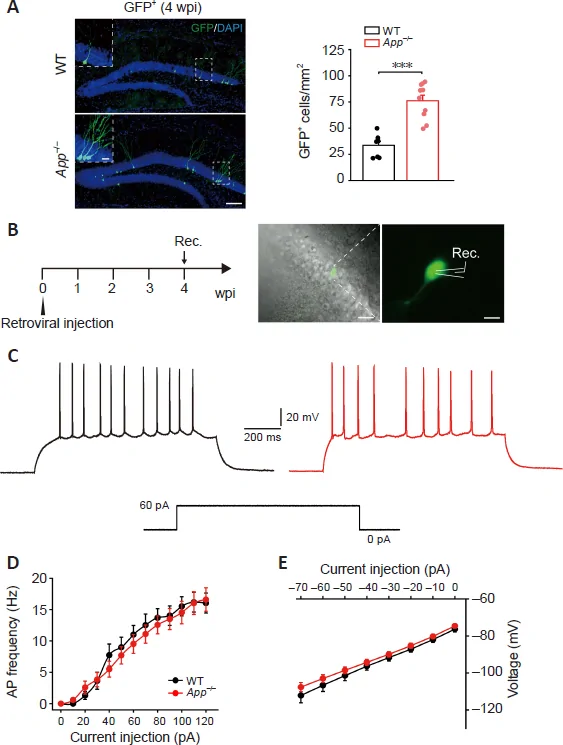
Increased number but unchanged intrinsic excitability of 4-week-old abGCs in *App*^–/–^ mice. (A) Representative images of neurons expressing retroviral GFP in the DG of WT (black) and *App*^–/–^ (red) mice. Scale bar: 100 µm. For enlarged images of areas in the dotted rectangles, scale bar: 20 µm. Quantitative analysis revealed that the number of GFP^+^ neurons in the DG significantly increased (*n* = 8 and 10 slices from WT and *App*^–/–^ mice, respectively). (B) Illustration of the experimental procedure (left); the fluorescent cell on the right is the recorded cell. Scale bar: 80 µm. For enlarged images of areas where the recorded cell resides, scale bars: 13 µm. (C) Representative AP traces evoked by injection of the depolarizing current (+60 pA). (D) Similar numbers of APs in response to current injections ranging from 0 to +120 pA in steps of 10 pA in the two groups (*P* = 0.740, *n* = 23 neurons from 7 WT mice and *n* = 30 neurons from 6 *App*^–/–^ mice). (E) Unchanged current‒voltage characteristic curve of abGCs (*P* = 0.340, *n* = 23 neurons from 7 WT mice, and *n* = 30 neurons from 6 *App*^–/–^ mice). Data are presented as the mean ± SEM. ***P < 0.001 (Student’s *t*-test [A] and two-way repeated-measures analysis of variance [D, E]). abGCs: Adult-born granule cells; AP: action potential; App: amyloid precursor protein; DG: dentate gyrus; GFP: green fluorescent protein; Rec.: record; wpi: week post-injection; WT: wild-type.

Although the effects of the environment on the survival and integration of abGCs are restricted to the first 3 weeks after their birth (Tashiro et al., 2007), 4-week-old abGCs are more plastic than neurons at other stages (Gu et al., 2012). To explore whether *App* deletion impacts 4-wpi abGCs, we injected the RVP-GFP construct into the hippocampus of *App*^−/−^ mice to generate abGCs expressing EGFP. Using fixed tissues, the number of 4-wpi abGCs in the DG was significantly higher in *App*^−/−^ mice than in WT mice (*P* < 0.001; **Figure 1A**). Whole-cell patch-clamp recordings of 4-wpi abGCs in acute brain slices revealed a comparable AP firing rate between the two genotypes (**Figure 1B–D**). Additionally, current injection-induced membrane potentials in 4-wpi abGCs did not differ significantly between *App*^−/−^ and WT mice (**Figure 1E**). These findings suggested that the absence of *App* increases the number of 4-wpi abGCs without altering their intrinsic excitability.

Upon entering the endoplasmic reticulum, APP traffics through the trans-Golgi network to the neuronal surface (Brunholz et al., 2012), where it interacts with specific membrane proteins to regulate neuronal properties (Sanchez-Alavez et al., 2007; Chen et al., 2017). To explore whether *App* deletion alters the membrane properties of 4-wpi abGCs, we applied gradually increasing short-duration depolarizing current injections and analyzed the first single AP. In *App*^−/−^ mice, abGCs exhibited significantly increased AP half-width and decay time (*P* < 0.05), while rise time remained unchanged (**Table 1**), suggesting immature electrophysiological properties in 4-wpi abGCs lacking APP.

**Table 1:** Intrinsic properties of adult-born granule cells at 4 weeks post-injection

Intrinsic property	WT	*n*	*App*–/–	*n*	*P*
Membrane capacitance (pF)	30.2	54 (11)	30.58	65 (10)	0.858
Input resistance (MΩ)	460	42 (11)	449	42 (10)	0.816
Resting membrane (mV)	–74.4	46 (11)	–74.4	44 (10)	0.210
Spiking threshold (mV)	–38.6	39 (11)	–37.5	40 (10)	0.365
AP amplitude (mV)	78.5	39 (11)	78.4	40 (10)	0.956
After-hyperpolarization (mV)	–6.85	28 (11)	–6.27	31 (10)	0.381
AP half-width (ms)	1.61	39 (11)	1.79	40 (10)	0.045
AP rise time (ms)	0.48	39 (11)	0.47	40 (10)	0.776
AP decay time (ms)	1.48	39 (11)	1.76	40 (10)	0.015

AP: Action potential; App: amyloid precursor protein; WT: wild-type.

**Figure 2:**
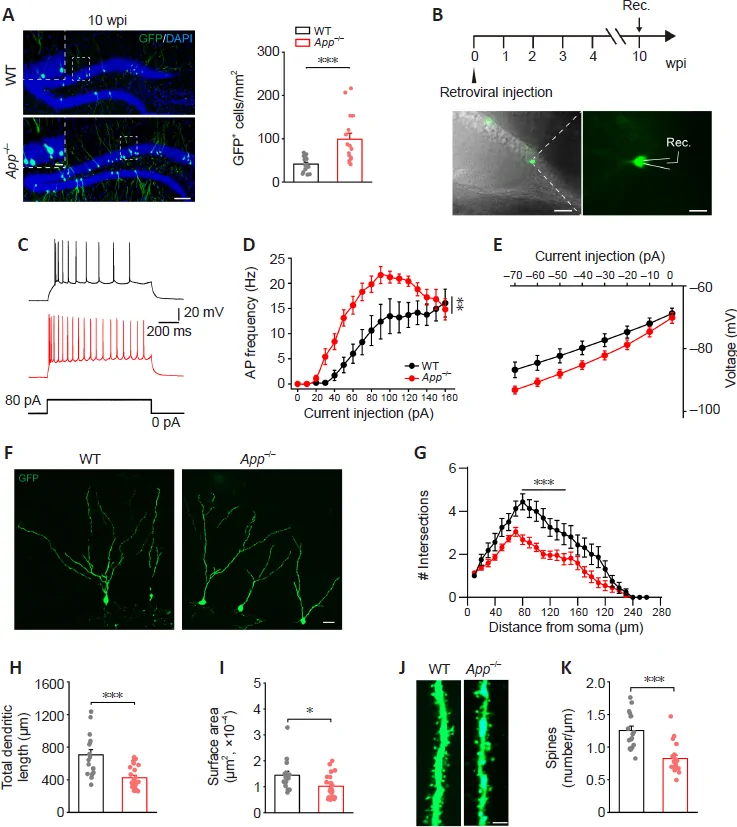
Increased number and intrinsic excitability of 10-week-old abGCs in the hippocampal DG of *App*^–/–^ mice. (A) Representative images of neurons expressing retroviral GFP in the DGs of WT (black) and *App*^–/–^ (red) mice. Scale bar: 100 µm. For enlarged images of areas in the dotted rectangles, the scale bar = 20 µm. Quantitative analysis revealed that the number of GFP^+^ neurons in the DG significantly increased (*n* = 19 slices from six WT mice and 16 slices from five *App*^–/–^ mice). (B) Illustration of the experimental procedure (left); the fluorescent cells on the right are the recorded cells. Scale bar: 80 µm. For enlarged images of areas where the recorded cell resides, scale bars: 24 µm. (C) Representative AP traces evoked by injection of depolarizing current (+80 pA). (D) Ten-week-old abGCs from *App*^–/–^ mice presented a greater number of APs in response to current injections ranging from 0 to +160 pA in steps of 10 pA (*P* < 0.01, *n* = 13 neurons from 3 WT mice and *n* = 17 neurons from 3 *App*^–/–^ mice). (E) Similar current‒voltage characteristic curves of abGCs in WT and *App*^–/–^ mice (*n* = 13 neurons from three WT mice and *n* = 17 neurons from three *App*^–/–^ mice). (F) Representative confocal images of RV-GFP-labeled abGCs at 10 wpi (green) in the dentate gyrus of WT and *App*^–/–^ mice. Scale bar: 20 µm. (G–I) Quantitative analysis revealed that APP deficiency caused dysfunction in the dendritic development of abGCs at 10 wpi (WT, *n* = 16 abGCs from three mice; *App*^–/–^, *n* = 22 abGCs from three mice. Two-way analysis of variance, genotype effect: *F*_(1, 936)_ = 114.100, *P* < 0.001; distance effect: *F*_(25, 936)_ = 35.650, *P* < 0.001; interaction: *F*_(25, 936)_ = 2.163, *P* < 0.001; total dendritic length: Student’s *t*-test, *t*_(36)_ = 4.255, *P* < 0.001; surface area: Student’s *t*-test, *t*_(36)_ = 2.492, *P* < 0.05). (J) Representative confocal images of tertiary apical dendrites from RV-GFP-labeled abGCs at 10 wpi (green) in the dentate gyrus of WT and *App*^–/–^ mice. Scale bar: 2 µm. (K) Quantitative analysis revealed that the dendritic spine density of RV-GFP-labeled abGCs at 10 wpi decreased in *App*^–/–^ mice (WT, *n* = 17 abGCs from three mice; *App*^–/–^, *n* = 20 abGCs from three mice; two to four segments/cell were chosen for counting; Student’s *t*-test, *t*_(35)_ = 5.203). Data are presented as the mean ± SEM. *P < 0.05, **P < 0.01, ***P < 0.001. abGCs: Adult-born granule cells; App: amyloid precursor protein; DG: dentate gyrus; GFP: green fluorescent protein; wpi: week post-injection; WT: wild-type.

It is widely accepted that abGCs require 6–8 weeks to mature, at which point their electrophysiological properties resemble those of mGCs that were produced embryonically or early postnatally (Laplagne et al., 2006; Toni and Schinder, 2015; Miller and Sahay, 2019; Cole et al., 2020). Furthermore, immature abGCs are thought to be electrophysiologically distinct from their mature counterparts (Toda et al., 2019). Given prior evidence that *App* deletion impairs the survival of newborn neurons, as evaluated by the ratio of 10-wpi abGCs to 4-wpi abGCs (Wang et al., 2014), we investigated whether 10-wpi abGCs from *App*^−/−^ mice display altered electrical properties that could contribute to their reduced survival. Consistent with the findings at 4 wpi, the number of 10-wpi abGCs in the DG was significantly higher in *App*^−/−^ mice than in WT mice (**Figure 2A**; *P* < 0.001). However, unlike 4-wpi abGCs, 10-wpi abGCs exhibited a significantly elevated AP firing rate in *App*^−/−^ mice (*P* < 0.01; **Figure 2B–D**), suggesting that the absence of APP increased neuronal excitability of abGCs at this stage. Current-injection-induced membrane potentials remained similar in the two groups (**Figure 2E**), and the spiking threshold did not differ. Notable, rheobase, a measure of membrane potential excitability (Matthews et al., 2009; Jaffe and Brenner, 2018), was significantly lower in *App*^−/−^ mice (**Table 2**), further supporting increased excitability. In line with the positive correlation between AP firing rate and input resistance (Amatrudo et al., 2012), membrane analysis showed that *App*^−/−^ 10-wpi abGCs showed significantly higher input resistance than WT cells (*P* < 0.05; **Table 2**). Furthermore, AP half-width, rise time, and decay time were all significantly prolonged (*P* < 0.05; **Table 2**), suggesting that abGCs from *App*^−/−^ mice retain immature electrical properties even at 10-wpi.

**Table 2:** Intrinsic properties of adult-born granule cells at 10 weeks post-injection

Intrinsic property	WT	*n*	*App*–/–	*n*	*P*
Membrane capacitance (pF)	24.9	12 (3)	29.7	15 (3)	0.189
Rheobase (pA)	96.7	12 (3)	60.7	15 (3)	0.0005
Input resistance (MΩ)	255	12 (3)	324	15 (3)	0.026
Resting potential (mV)	–70.7	12 (3)	–69.4	15 (3)	0.502
Spiking threshold (mV)	–41.2	12 (3)	–39.9	15 (3)	0.792
AP amplitude (mV)	76.6	12 (3)	78.9	15 (3)	0.151
After-hyperpolarization (mV)	–5.05	12 (3)	–5.17	15 (3)	0.916
AP half-width (ms)	1.4	12 (3)	1.54	15 (3)	0.044
AP rise time (ms)	0.43	12 (3)	0.48	15 (3)	0.020
AP decay time (ms)	1.27	12 (3)	1.46	15 (3)	0.023

AP: Action potential; App: amyloid precursor protein; WT: wild-type.

During maturation, abGCs interact dynamically with the pre-existing network, altering their intrinsic and synaptic properties to reach morphological and functional maturity (Groisman et al., 2020). This involves extensive dendritic remodeling, including morphological changes in dendritic spines (Groisman et al., 2020; Baczynska et al., 2021; Denoth-Lippuner and Jessberger, 2021). To evaluate abGC maturity, we examined dendritic morphology at 10-wpi in *App*^−/−^ and WT mice. Analysis of RV-GFP-labeled abGCs (**Figure 2F**) revealed a significant reduction in the number of dendritic intersections in *App*^−/−^ mice (*P* < 0.001; **Figure 2G**), along with decreased total dendritic length (*P* < 0.001; **Figure 2H**), dendritic area (*P* < 0.05; **Figure 2I**), and spine number on dendrites (*P* < 0.001; **Figure 2J** and **K**).

### Miniature synaptic transmission of adult-born granule cells at 4 and 10 weeks post-injection

**Figure 3:**
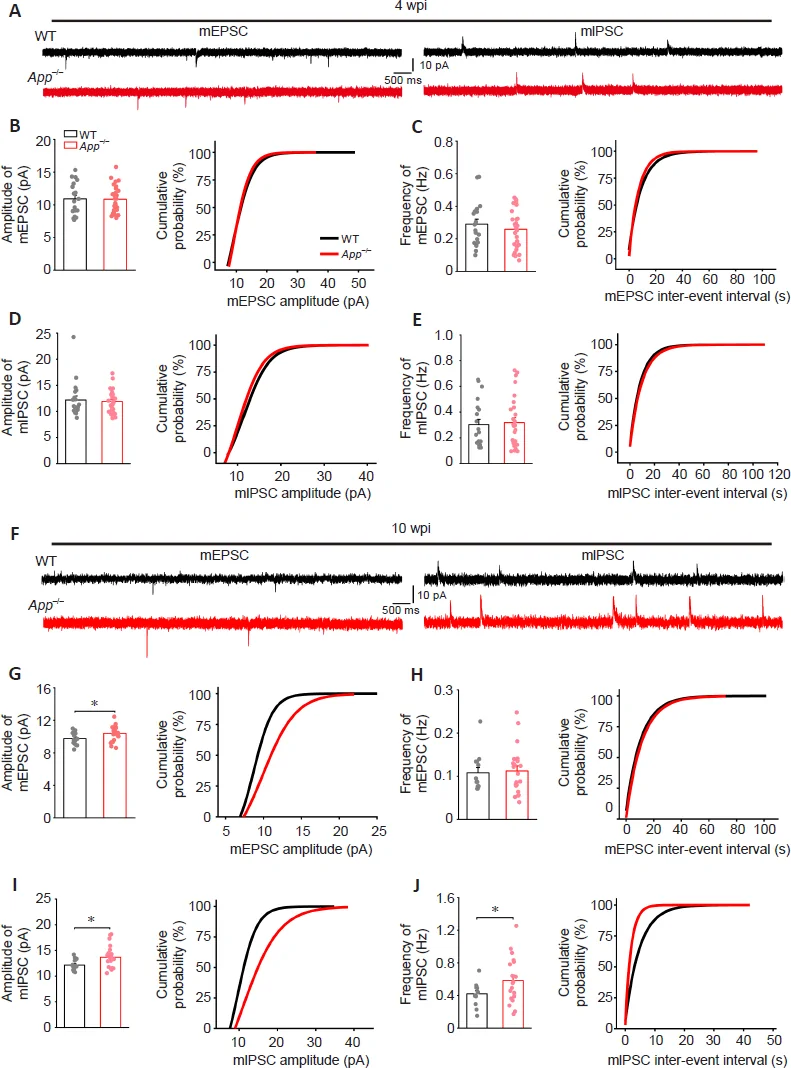
Increased excitatory and inhibitory synaptic transmission of 10-wpi abGCs, but not 4-wpi abGCs, in *App*^–/–^ mice. (A–E) Unchanged mEPSCs and mIPSCs in 4-wpi abGCs (*n* = 20 neurons from eight WT (black) mice and *n* = 29 neurons from eight *App*^–/–^ (red) mice) (mEPSC: amplitude: *P* = 0.936; frequency: *P* = 0.409) (mIPSC: amplitude: *P* = 0.584; frequency: *P* = 0.788). (F) Representative mEPSC and mIPSC traces of 10-wpi abGCs. (G, H) The frequency but not the amplitude of mEPSCs was significantly increased in *App*^–/–^ mice (amplitude: *P* = 0.808; frequency: *P* < 0.05) (*n* = 14 neurons from three WT mice; *n* = 21 neurons from three *App*^–/–^ mice). (I, J) Increased amplitude and frequency of mIPSCs in *App*^–/–^ mice (amplitude: *P* < 0.05; frequency: *P* < 0.05) (*n* = 14 neurons from three WT mice and *n*=20 neurons from three *App*^–/–^ mice). Data are presented as mean ± SEM. *P < 0.05 (Student’s *t*-test). abGCs: Adult-born granule cells; App: amyloid precursor protein; mEPSC: miniature excitatory postsynaptic current; mIPSC: miniature inhibitory postsynaptic current; wpi: week post-injection; WT: wild-type.

During the window between 4 and 6 weeks post birth, abGCs begin integrating into the hippocampal neural network, becoming similar to mGCs by 7 weeks (Toni and Schinder, 2015). To explore whether *App* deletion impacts synaptic transmission in immature abGCs, we recorded whole-cell miniature postsynaptic currents in 4-wpi abGCs, but found no significant difference between *App*^−/−^ and WT mice. Both amplitude and frequency of mEPSCs (**Figure 3A–C**) and mIPSCs (**Figure 3A**, **D**, and **E**) were comparable between the groups, indicating that *App* deletion does not alter synaptic transmission at 4 wpi.

In contrast, 10-wpi abGCs from *App*^−/−^ mice exhibited a significantly increased mEPSC amplitude (**Figure 3F** and **G**), while mEPSC frequency remained unchanged (**Figure 3F** and **H**), suggesting enhanced excitatory synaptic activity via postsynaptic mechanisms. Concurrently, both the amplitude (*P* < 0.05; **Figure 3F** and **I**) and frequency (*P* < 0.05; **Figure 3F** and **J**) of mIPSCs were significantly elevated, implying that *App* deletion alters inhibitory synaptic transmission through both pre- and postsynaptic mechanisms in 10-wpi abGCs.

### Action potential firing, membrane properties, and miniature synaptic transmission of mature granule cells

**Figure 4:**
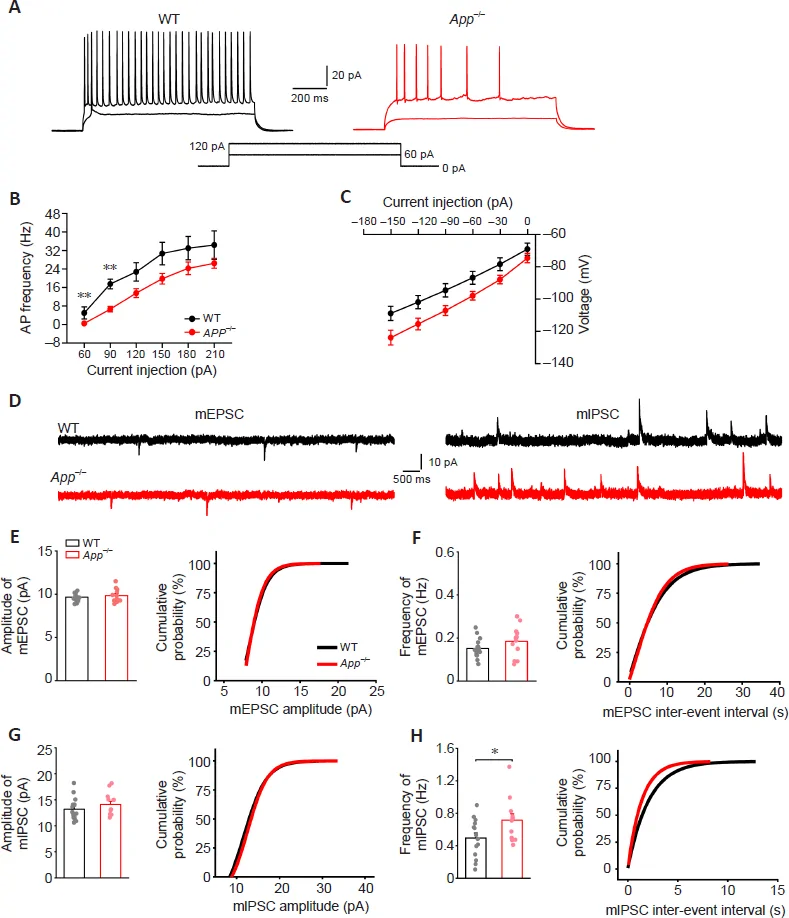
Reduced intrinsic excitability and increased mIPSC frequency of mGCs in *App*^–/–^ mice. (A) Representative AP traces evoked by injection of depolarizing currents (+120 pA and +60 pA) in the DGs of WT (black) and *App*^–/–^ (red) mice. (B) mGCs of *App*^–/–^ mice exhibit a lower number of APs in response to current injections ranging from +60 to +210 pA in steps of 30 pA (60 pA: *P* < 0.01; 90 pA: *P* < 0.01, *n* = 12 neurons from four WT mice, and *n* = 27 neurons from six *App*^–/–^ mice). (C) Current–voltage characteristic curves of mGCs in WT and *APP*^–/–^ mice (*n* = 12 neurons from four WT mice and *n* = 27 neurons from six *App*^–/–^ mice: *P* = 0.054). (D) Representative mEPSC and mIPSC traces of mGCs. (E, F) Identical mEPSC amplitude and frequency in *App*^–/–^ mice (amplitude: *P* = 0.429; frequency: *P* = 0.149; *n* = 14 neurons from three WT mice; *n* = 12 neurons from three *App*^–/–^ mice). (G, H) The frequency but not the amplitude of mIPSCs was significantly increased in *App*^–/–^ mice (amplitude: *P* = 0.808; frequency: *P* < 0.05, *n* = 14 neurons from three WT mice, and *n* = 12 neurons from three *App*^–/–^ mice). Data are presented as mean ± SEM. *P < 0.05, **P < 0.01 (repeated-measures analysis of variance [B], Student’s *t*-test [E–H]). App: Amyloid precursor protein; mEPSCs: miniature excitatory postsynaptic currents; mGCs: mature granule cells; mIPSCs: miniature inhibitory postsynaptic currents; WT: wild-type.

Having shown that *App* deletion causes hyperexcitability and enhances both excitatory and inhibitory synaptic transmission in 10-wpi abGCs, we next examined whether similar effects occur in mGCs born prior to RV-GFP injection and thus older than 10 weeks. We noted that, unlike 10-wpi abGCs, mGCs from *App*^−/−^ mice exhibited a significantly lower AP firing rate compared with WT mice (**Figure 4A** and **B**), while current-injection-induced membrane potentials remained unchanged (**Figure 4C**). This indicated that, in contrast to 10-wpi abGCs, mGCs experience reduced excitability, with APP deficiency initially increasing and later decreasing intrinsic excitability with age.

Consistent with reduced AP firing, *App*^−/−^ mGCs displayed a significantly higher AP firing threshold compared with WT counterparts (*P* < 0.05; **Table 3**), indicating a requirement for greater depolarization to elicit action potentials. Additionally, *App*^−/−^ mGCs exhibited elevated after-hyperpolarization (*P* < 0.05; **Table 3**), in line with previous findings of reduced neuronal excitability (Matthews et al., 2009; Jaffe and Brenner, 2018).

**Table 3:** Intrinsic properties of mature granule cells

Intrinsic property	WT	*n*	*App*–/–	*n*	*P*
Membrane capacitance (pF)	72	12 (4)	66	27 (6)	0.060
Input resistance (MΩ)	269	12 (4)	316	27 (6)	0.430
Resting potential (mV)	–70	12 (4)	–74	27 (6)	0.170
Spiking threshold (mV)	–42	12 (4)	–32	27 (6)	0.030
AP amplitude (mV)	62	12 (4)	61	27 (6)	0.430
After-hyperpolarization (mV)	2.6	12 (4)	9.4	27 (6)	0.001
AP half-width (ms)	1.34	12 (4)	1.61	27 (6)	0.150
AP rise time (ms)	0.45	12 (4)	0.59	27 (6)	0.300
AP decay time (ms)	1.01	12 (4)	1.17	27 (6)	0.260

AP: Action potential; App: amyloid precursor protein; WT: wild-type.

Miniature synaptic transmission analysis of mGCs revealed no significant differences in the amplitude (**Figure 4D** and **E**) or frequency (**Figure 4D** and **F**) of mEPSCs between genotypes. In mIPSCs, the amplitude was also unchanged (**Figure 4D** and **G**), but the frequency was significantly increased in *App*^−/−^ mice (**Figure 4D** and **H**).

In summary, electrophysiological abnormalities were most pronounced in 10-wpi abGCs of *App*^−/−^ mice. At 4 wpi, *App*^−/−^ abGCs showed altered AP half-width and decay time, but no changes in firing rate or synaptic transmission. By 10 wpi, abGCs exhibited elevated AP firing rate, increased input resistance, reduced AP firing threshold, and prolonged AP kinetics (upregulated half-width, rise time, and decay time). Synaptically, 10-wpi abGCs exhibited increased mEPSC and mIPSC amplitudes and elevated mIPSC frequency. In contrast, *App*^−/−^ mGCs displaced reduced AP firing, increased firing threshold, and elevated mIPSC frequency. These findings highlighted a crucial role for APP in regulating the electrical properties of GCs, with distinct effects on immature versus mature neurons.

### Potassium-chloride-cotransporter 2 expression and apoptosis in the dentate gyrus

**Figure 5:**
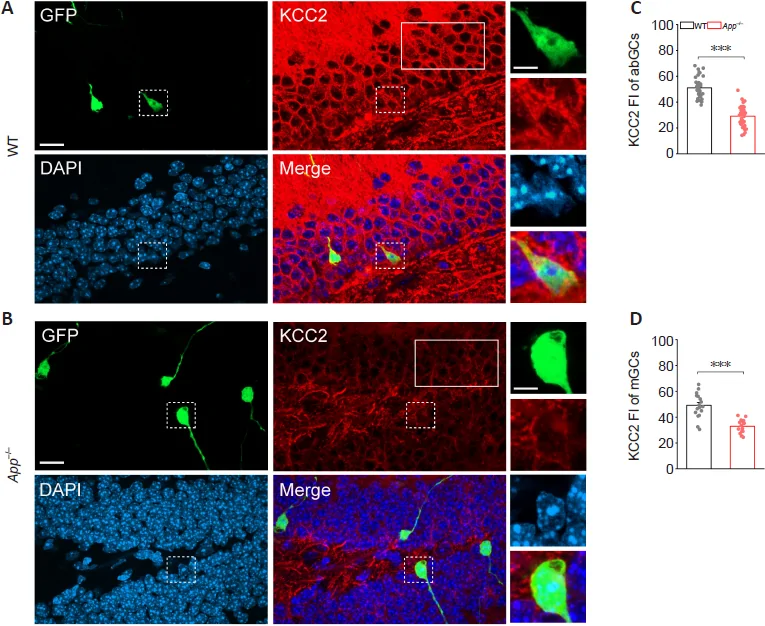
Decreased KCC2 levels in 10-wpi abGCs and mGCs in the *App*-deficient DG. (A, B) Representative confocal images of RV-GFP-labeled abGCs at 10 wpi (green) in the DG of WT (black) and *App*^–/–^ (red) mice showing KCC2 in red and DAPI (chromatin) in blue. Scale bar: 20 µm. The insets on the right show enlarged images of the regions in the dotted squares showing the GFP-labeled abGCs. Scale bar: 5 µm. The KCC2 fluorescence intensity (FI) within the solid line box was quantified to determine KCC2 immunoreactivity in mGCs. (C, D) Quantitative analysis indicating that KCC2 levels in both abGCs at 10 wpi and mGCs are significantly lower in the DGs of *App*^–/–^ mice than in those of WT controls (mean ± SEM; KCC2 immunoreactivity of abGCs: *n* = 37 abGCs from three mice per group; Student’s *t*-test: *t*_(72)_ = 11.610, *P* < 0.001; KCC2 immunoreactivity of mGCs: *n* = 18 views from three mice per group; Student’s *t*-test, *t*_(34)_ = 6.379, *P* < 0.001). ***P < 0.001. abGCs: Adult-born granule cells; App: amyloid precursor protein; DAPI: 4′,6-diamidino-2-phenylindole; DG: dentate gyrus; GFP: green fluorescent protein; KCC2: potassium-chloride-cotransporter 2; mGCs: mature granule cells; wpi: week post-injection; WT: wild-type.

The neuron-specific potassium-chloride transporter, KCC2, is critical for the dynamic control of GABA signaling and guiding the transition of abGCs from immature to mature states. Immature neurons express low levels of KCC2 and maintain high intracellular Cl^−^ concentrations, resulting in an inverted Cl^−^ potential and an initially depolarizing effect of GABA on the membrane potential of abGCs (Ben-Ari, 2002; Blaesse et al., 2009). On maturation, KCC2 expression increases, promoting Cl^−^ extrusion and maintaining low intracellular Cl^−^ homeostasis, consequently abrogating their high input resistance (Ge et al., 2006; Markwardt et al., 2009; Bergami et al., 2015). We previously reported that KCC2 levels are significantly reduced in the hippocampus of *App*^−/−^ mice (Chen et al., 2017). Here, we evaluated KCC2 levels in 10-wpi mGCs and abGCs (**Figure 5A** and **B**) and found significantly decreased levels in both cell types in *App*^−/−^ mice compared with those in WT mice (*P* < 0.001; **Figure 5C** and **D**).

**Figure 6:**
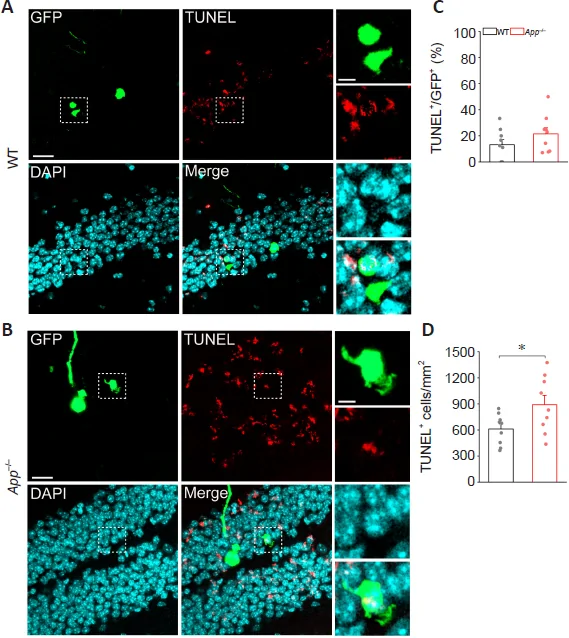
Increased levels of TUNEL in 10-wpi abGCs in the APP-deficient DG. (A, B) Representative confocal images of RV-GFP-labeled abGCs at 10 wpi (green) showing TUNEL in red and DAPI (chromatin) in blue in the DGs of WT and *App*^–/–^ mice. Scale bar: 20 µm. The insets on the right are enlarged images of the regions in the dotted squares showing the GFP-labeled abGCs. Scale bar: 5 µm. (C, D) Quantitative analysis revealed an increase in the number of TUNEL^+^ cells in the DG of *App*^–/–^ (red) mice compared with WT (black) mice, and the percentage of TUNEL^+^-GFP^+^ cells/GFP^+^ cells was unchanged (means ± SEMs; percentage of TUNEL^+^-GFP^+^ cells/GFP^+^ cells: *n* = 9 slices from three mice per group; Student’s *t*-test, *t*_(16)_ = 1.357, *P* = 0.194; TUNEL^+^ cells/mm^2^: *n* = 9 slices from three mice per group; Student’s *t*-test, *t*_(16)_ = 2.281, *P* < 0.05). *P < 0.05. abGCs: Adult-born granule cells; App: amyloid precursor protein; DAPI: 4′,6-diamidino-2-phenylindole; DG: dentate gyrus; GFP: green fluorescent protein; KCC2: potassium-chloride-cotransporter 2; mGCs: mature granule cells; TUNEL: terminal deoxynucleotidyl transferase; dUTP nick-end labeling; wpi: week post-injection; WT: wild-type.

Most abGCs undergo programmed cell death (apoptosis) within the week following division, a period that coincides with synaptogenesis and is marked by the loss of a substantial number of immature neurons, even under physiological conditions. Mature neurons also undergo spontaneous apoptosis to maintain a dynamic balance in the adult brain (Sierra et al., 2010; Ryu et al., 2016). The critical survival period for abGCs is within the first 4 weeks post birth, and 4-week old abGCs comprise less than 1% of the total GC population (Dieni et al., 2012). Following maturation, they become resistant to cell death (Vivar et al., 2012; Deshpande et al., 2013; Kaila et al., 2014). To assess apoptosis in 10-wpi abGCs and mGCs from *App*^−/−^ mice, we used the TUNEL assay, which detects cell death-related DNA fragmentation (Darzynkiewicz et al., 2008; Moore et al., 2021). *App*^−/−^ mGCs exhibited significantly increased TUNEL labeling compared with WT mGCs (*P* < 0.05; **Figure 6A–D**), indicating elevated apoptosis. Notably, in addition to regulating intracellular Cl^−^ homeostasis (Ben-Ari, 2002; Kaila et al., 2014), KCC2 supports neuronal survival by limiting apoptosis (Kontou et al., 2021). Thus, deficient KCC2 expression may contribute, at least in part, to the developmentally increased apoptosis observed in *App*^−/−^ GCs.

### Parvalbumin-positive interneuron abundance and intrinsic activity in the dentate gyrus

**Figure 7:**
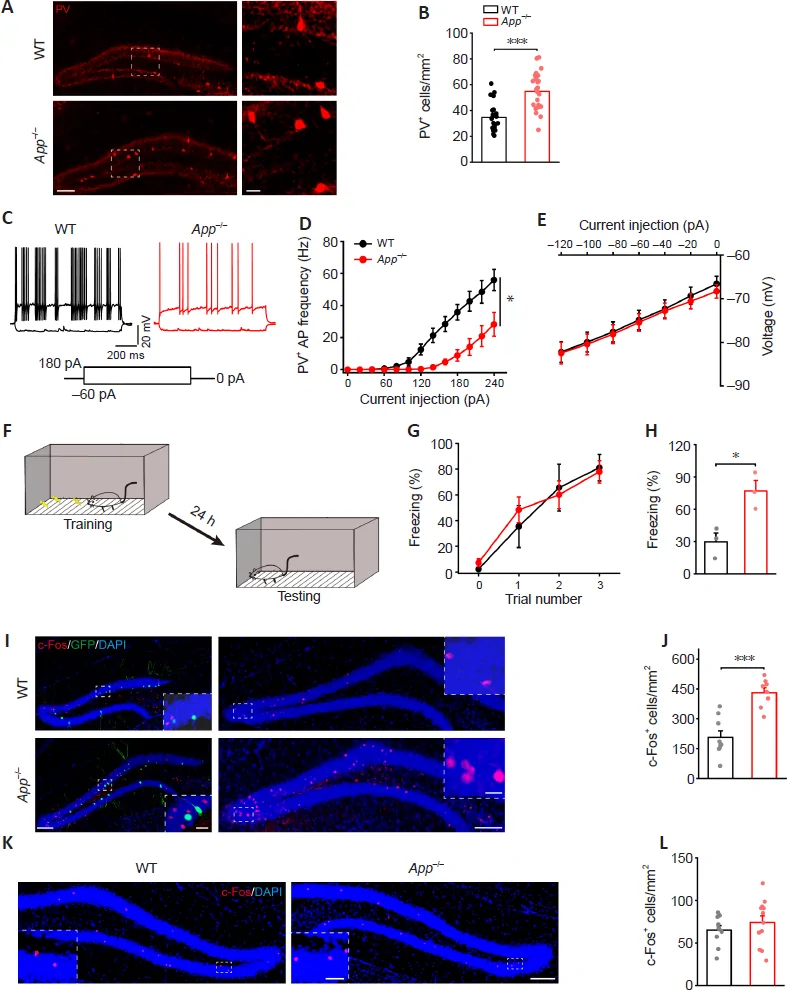
Reduced excitability of PV^+^ interneurons and increased levels of freezing behavior and number of fear memory-induced c-Fos-positive cells in *App*^–/–^ mice. (A) Representative images of PV^+^ interneurons in the DG of WT (black) and *App*^–/–^ (red) mice. Scale bar: 100 µm. For enlarged images of areas in the dotted rectangles, scale bar: 20 µm. (B) Quantitative analysis revealed that the number of PV^+^ neurons in the *App*^–/–^ DG significantly increased (*n* = 23 slices from five WT mice and 21 slices from five *App*^–/–^ mice, *P* < 0.001). (C) Representative AP firing in PV^+^ interneurons. (D) Reduced AP firing in PV^+^ interneurons in the *App*^–/–^ DG (*P* < 0.05, *n* = 14 neurons from three WT mice, and *n* = 10 neurons from three *App*^–/–^ mice). (E) Current–voltage characteristic curves of PV^+^ interneurons in WT and *App*^–/–^ mice (*P* = 0.064, *n* = 14 neurons from three WT mice, and *n* = 10 neurons from three *App*^–/–^ mice; two-way repeated-measures analysis of variance). (F) Illustration of the CFC. (G) Freezing behavior was not changed during the training period (*P* = 0.875, *n* = 3 for WT mice, and *n* = 3 for *App*^–/–^ mice). (H) *App*^–/–^ mice exhibited significantly increased freezing behavior in the test period (*P* < 0.05; *n* = 3 for WT mice and *n* = 3 for *App*^–/–^ mice). (I) Representative images of c-Fos-positive cells in the DG of WT and *App*^–/–^ mice after CFC. (J) Increased numbers of c-Fos-positive cells in *App*^–/–^ mice after fear memory. There was no overlap of c-Fos- and retroviral-GFP-positive cells (*n* = 9 slices from five WT mice and *n* = 8 slices from three *App*^–/–^ mice, *P* < 0.001). (K) Representative images of the basal number of c-Fos-positive cells in the DG of WT and *App*^–/–^ mice. (L) Quantification of c-Fos staining revealed similar numbers of c-Fos-positive cells (*n* = 10 slices from three WT mice and 12 slices from three *App*^–/–^ mice, *P* = 0.366). Scale bar: 100 µm. For enlarged images of areas in the dotted rectangles, scale bar: 20 µm. Data are presented as the mean ± SEM. *P < 0.05, ***P < 0.001 (Student’s *t*-test [B, H, J, L], two-way repeated-measures analysis of variance [D]). AP: Action potential; App: amyloid precursor protein; CFC: contextual fear conditioning; DG: dentate gyrus; PV: parvalbumin; TUNEL: terminal deoxynucleotidyl transferase dUTP nick-end labeling; WT: wild-type.

The abGCs initially receive synaptic input from local interneurons, followed by long-range projections of neurons from other brain regions (Vivar et al., 2012; Deshpande et al., 2013; Bergami et al., 2015). APP, highly expressed in the majority of GABAergic neurons in the DG, enhances inhibitory signaling to GCs (Wang et al., 2014). Considering that PV^+^ interneurons are the predominant GABAergic inhibitory subtype in the DG, and that GABAergic inhibition plays a critical role in regulating GC activity (Song et al., 2012; Alvarez and Giacomini, 2016; Pelkey et al., 2017) and DG-involved memory (Zou et al., 2016), we sought to quantify PV^+^ interneurons via immunofluorescent staining using an anti-PV antibody. Compared with WT mice, we observed a significant increase in the number of PV^+^ interneurons in the DG of *App*^−/−^ mice (*P* < 0.001; **Figure 7A** and **B**), consistent with the increased mIPSC frequency observed in 10-wpi abGCs and mGCs. Notably, despite their increased numbers, PV^+^ interneurons exhibited significantly decreased intrinsic excitability, as indicated by markedly reduced AP firing, in the DG of *App*^−/−^ mice (*P* < 0.05; **Figure 7C** and **D**), while current-induced voltage remained unchanged (**Figure 7E**). These findings suggested that the reduced excitability of PV^+^ interneurons in *App*^−/−^ mice may impair presynaptic AP-triggered GABA release from axonal termini, thereby weakening GABAergic inhibition of GCs despite increased interneuron abundance.

### Expression of synaptic receptors and brain-derived neurotrophic factor/tropomyosin-related kinase receptor type B in the dentate gyrus

To investigate potential changes in excitatory and inhibitory synaptic receptors, we conducted immunofluorescence staining of the excitatory receptor AMPAR, the inhibitory receptor subunits GABA_A_Rα1 and GABA_A_Rα5, and the brain-derived neurotrophic factor (BDNF)/tropomyosin-related kinase receptor type B (TrkB) pathway—a key regulator of synaptic activity, neuronal survival, and structural plasticity (Huang and Reichardt, 2001; Binder and Scharfman, 2004). Compared with WT controls, AMPAR levels remained unchanged in the DG of *App*^−/−^ mice, whereas GABA_A_Rα1 and GABA_A_Rα5 levels were significantly reduced (**Additional Figure 1A–E**), indicating impaired inhibitory neurotransmission and an elevated excitation/inhibition (E/I) ratio. Although BDNF expression remained unchanged, its receptor TrkB was significantly upregulated in the *App*^−/−^ DG (**Additional Figure 2A–C**).

### Fear memory and abundance of c-Fos-positive cells in the dentate gyrus

*App*^−/−^ mice reportedly exhibit impairments in spatial memory (Dawson et al., 1999) and CFC (Wang et al., 2014). The abGCs contribute to specific forms of memory processing, including fear memory (Luna et al., 2019; Miller and Sahay, 2019), and 4- to 6-week-old abGCs are necessary for specific forms of hippocampal-dependent learning (Luna et al., 2019; Miller and Sahay, 2019). To explore whether changes in *App*^−/−^ abGCs affect fear memory, we conducted CFC testing (**Figure 7F**). During the adaptation phase, both *App*^−/−^ and WT mice showed similar freezing behavior (**Figure 7G**). However, during the test phase, *App*^−/−^ mice were significantly more likely to freeze (*P* < 0.05; **Figure 7H**). Given the involvement of DG-derived GCs in mediating fear conditioning (Kheirbek et al., 2012; Nakashiba et al., 2012; Miller and Sahay, 2019) and the requirement of c-Fos expression for fear memory (Gallo et al., 2018), we quantified c-Fos^+^ cells in the DG following CFC (Zhang et al., 2002). Brains from naive or CFC-tested mice (1.5 hours post-test) were perfused, sectioned, and stained with anti-c-Fos antibody. Compared with WT mice, *App*^−/−^ mice showed a significant increase in c-Fos^+^ cells following CFC (*P* < 0.001; **Figure 7I** and **J**). Notably, no cells with double-positivity for both c-Fos and RV-GFP were detected in the DG of either genotype (**Figure 7I**). Basal c-Fos levels were similar in WT and *App*^−/−^ mice (**Figure 7K** and **L**). Together, these results suggested that fear conditioning preferentially activates mGCs in *App*^−/−^ mice, while abGCs remain unengaged in both genotypes.

## Discussion

The findings of the present study reveal that APP deficiency alters the intrinsic excitability and synaptic transmission of abGCs in a cellular age-dependent manner. Of note, we show that 10-week-old *App*^−/−^ abGCs exhibit intrinsic and synaptic hyperexcitability, indicative of excitotoxicity associated with shortened dendrites, fewer intersections and spines, reduced KCC2 expression, and increased mGC apoptosis, despite a compensatory increase in abGC genesis. The marked increase in excitatory response observed in *App*^−/−^ abGCs at 10 wpi is consistent with excitotoxicity, an electrical characteristic driven by excessive glutamatergic activity that can lead to neuronal death (Bowles et al., 2021; Neves et al., 2023; Yu et al., 2023). Together with reduced KCC2 levels and increased TUNEL signals in mGCs, we reason that the increased excitotoxicity in 10-wpi abGCs contributes to enhanced neuronal apoptosis, due at least in part to decreased KCC2 expression. Upon maturation, this disrupts Cl⁻ homeostasis and weakens GABAergic inhibitory efficacy (Ben-Ari, 2002; Kahle et al., 2008; Chen et al., 2017), possibly explaining the reduced levels of mGCs in the APP-deficient DG. Additionally, the number of abGCs was increased, consistent with our previous finding of elevated BrdU^+^ neurons in the DG of *App*^−/−^ mice (Wang et al., 2014). This may reflect a compensatory mechanism aimed at counteracting excessive GC apoptosis, temporarily offsetting cell death while failing to rescue long-term neuronal dysfunction. Thus, in addition to characterizing the electrical properties of abGCs, our study elucidates molecular mechanisms underlying their defective maturation and activity under APP-deficient conditions.

It is generally believed that abGCs reach maturity by 2 months of age and become functionally equivalent to the broader neuronal population (Cole et al., 2020). Hyperexcitability and high membrane input resistance are characteristics of immature neurons (Toni and Schinder, 2015). Although immature abGCs receive less inhibitory and excitatory input than mature cells (Dieni et al., 2013), they respond to a broader range of stimuli (Mongiat et al., 2009) and are hyperexcitable (Mongiat et al., 2009; Schmidt-Hieber et al., 2004; Dieni et al., 2013). This study reveals that 10-wpi abGCs retain immature electrical and molecular properties in the absence of APP, as evidenced by increased intrinsic excitability, elevated membrane input resistance, and reduced rheobase and KCC2 levels, collectively suggesting delayed maturation. Furthermore, the divergent effects of *App* deletion—inducing hyperexcitability in 10-wpi abGCs *versus* hypoexcitability in mGCs—likely reflect their distinct developmental origins and maturation timelines (Kempermann, 2015), pointing to impaired progression through neuronal maturation stages. While mGCs originate prenatally, abGCs undergo prolonged postnatal maturation (4–10 weeks) (Toni et al., 2008), making them to vulnerable to APP deficiency during this critical developmental window and resulting in abnormal Cl⁻ homeostasis. KCC2 serves as a key maturation marker, with its developmental upregulation converting GABAergic responses from depolarizing (immature) to hyperpolarizing (mature) states (Rivera et al., 1999; Ben-Ari, 2002). Reduced KCC2 expression in *App*^−/−^ mice indicates impaired neuronal maturation and compromised GABAergic inhibition, ultimately contributing to neuronal hyperexcitability (Kahle et al., 2008; Chen et al., 2017). Furthermore, KCC2 interacts with cytoskeletal proteins (e.g., 4.1N, Ankyrin-G) via its C-terminal domain to facilitate dendritic branching and synaptogenesis (Li et al., 2007; Gauvain et al., 2011). KCC2 deficiency consequently leads to impaired dendritic arborization and reduced spine density. The increased TrkB levels likely represent a compensatory response to deficient spine development, consistent with the established role of the BDNF/TrkB pathway in dendritic regulation (Colucci-D’Amato et al., 2020; Wang et al., 2022). Therefore, intrinsic hyperexcitability, increased miniature synaptic activities, and reduced KCC2 expression in 10-wpi abGCs support the conclusion that abGC maturation is impaired in the absence of APP. Considering that increased neuronal excitability can enhance subthreshold connectivity and amplify the activity of existing synapses, hyperexcitable 10-wpi abGCs may partially compensate for the reduced excitability of mGCs in *App*^−/−^ mice.

During defined windows of time, abGCs undergo experience-dependent survival and receive innervation from excitatory and inhibitory neurons, recruiting GABAergic interneurons at elevated rates (Epp et al., 2007; Tashiro et al., 2007; Bergami et al., 2015). The abGCs receive GABAergic input from local interneurons and form GABAergic synapses with features characteristic of immature connections (Overstreet Wadiche et al., 2005). GABAergic neurons express high levels of APP, which has been proposed to control the genesis and survival of abGCs via inhibitory signaling (Wang et al., 2014). vGAT-Cre and NEX-Cre mice were used to conditionally delete *App* in GABAergic and glutamatergic neurons, respectively, this approach was combined with BrdU, SOX2, DCX (an immature neuron marker), and NeuN (a mature neuron marker) labeling to trace neurogenesis. Their findings demonstrated that APP regulates abGCs in a non-cell-autonomous manner through GABAergic interneurons. In this study, we found that PV^+^ interneurons exhibited significantly reduced intrinsic excitability along with reduced levels of GABA_A_R expression, suggesting decreased PV^+^-mediated GABAergic inhibitory tone in the DG of *App*^−/−^ mice. Given that spontaneous miniature synaptic potentials arise from vesicle pools distinct from those released by AP-driven neural activity (Zucker, 2005), the increased number of PV^+^ interneurons may contribute to the elevated mIPSC frequency observed in abGCs and mGCs. Concurrently, decreased GABAergic inhibition is also supported by reduced KCC2 expression, which determines the strength and polarity of GABAergic inhibition (Ben-Ari, 2002; Chen et al., 2017). We also observed increased apoptosis in mGCs from *App*^−/−^ mice. Beyond its role in Cl^−^ homeostasis, KCC2 is essential for neuronal survival, preventing calcium overload, reducing mitochondrial damage, and suppressing caspase activation (Kontou et al., 2021). Conversely, KCC2 deficiency induces neuronal hyperexcitation, ultimately leading to cell death (Cheng et al., 2001; Sekerdag et al., 2018). Therefore, we reason that APP deficiency downregulates KCC2, contributing to neuronal hyperexcitability (Sivakumaran et al., 2015; Raol et al., 2020) and subsequent neuronal apoptosis.

The abGCs play an important role in behavior during their immature phase (McHugh et al., 2022; Arribas et al., 2023; Tuncdemir et al., 2023). Chronic ablation or genetic disruption of abGC synaptic plasticity impairs contextual fear memory in mice (Kheirbek et al., 2012; Miller and Sahay, 2019). Conversely, expansion of 5- to 8-week-old abGCs improves water maze performance, while genetically enhancing abGC survival during development improves discrimination between two similar environments (Sahay et al., 2011; Frechou et al., 2024). We observed upregulated c-Fos levels in activated mGCs following fear conditioning, possibly reflecting compensatory recruitment due to insufficient excitability of individual mGCs in *App*^−/−^ mice. Furthermore, deficient PV^+^ interneuron-mediated inhibition in vivo may lead to mGC overactivation, impairing pattern separation and increasing fear generalization (Linder and Burr, 1988; Kheirbek et al., 2013; Besnard and Sahay, 2016). Although abGCs are critically involved in cognition, including fear memory (Huckleberry et al., 2018; Besnard and Sahay, 2021), our findings indicate that 10-wpi abGCs from 3- to 4-month old mice were not involved in mediating fear conditioning under the present protocol, in either WT or *App*^−/−^ mice, highlighting their functional limitations in this behavioral context. Therefore, in addition to revealing the role of APP in DG neurogenesis and maturation, this research also provides new insights into its broader contribution to hippocampal function.

This study has several limitations that should be noted. First, the exclusive use of *App*^−/−^ mice precludes differentiation between the direct effects of APP loss-of-function and potential compensatory developmental adaptations. Future studies employing conditional or inducible *App* deletion in adult neurogenic niches could help to clarify cell-autonomous versus non-cell-autonomous mechanisms. Second, our electrophysiological assessments were conducted *ex vivo* using acute brain slices, which may not fully recapitulate the dynamic network activity and neuromodulatory influences present *in vivo*. Third, the lack of longitudinal tracking of individual abGCs limits direct causal links between the observed hyperexcitability at 10 wpi and subsequent apoptosis in mGCs. Finally, while CFC revealed behavioral differences and aberrant mGC activation, the functional contribution of abGCs to hippocampal-dependent behaviors remains undefined, warranting further exploration using abGC-specific manipulation strategies.

In conclusion, the present study demonstrates that APP deficiency disrupts the electrophysiological, morphological, and biochemical maturation of abGCs in an age-dependent manner. The excitotoxicity observed in abGCs and consequent mGC apoptosis may recapitulate early hippocampal dysfunction in AD, where neuronal hyperactivity precedes neurodegeneration (Busche et al., 2008; Palop and Mucke, 2010; Siskova et al., 2014). Collectively, our findings reveal a critical role for APP in regulating the generation and maturation of abGCs, and highlight adult neurogenesis as a potential therapeutic target for AD.

## Additional files:

***Additional Figure 1:*** *Levels of GABA*_*A*_*Rα1, AMPAR1 and GABA*_*A*_*Rα5 in the DGs of WT and App*^*–/–*^ *mice.*

Additional Figure 1 Levels of gamma-aminobutyric acid A receptor alpha 1 (GABA_A_Rα1), alpha-amino-3-hydroxy-5-methylisoxazole-4-propionate receptor (AMPAR1) and gamma-aminobutyric acid A receptor alpha 5 (GABA_A_Rα5) in the DGs of WT and *App*^-/-^ mice.
**(A)** Representative confocal images of GCs in the DG of WT (black) and APP^-/-^ (red) mice showing gamma-aminobutyric acid A receptor alpha 1 (GABA_A_Rα1) in red and DAPI (chromatin) in blue. Scale bar: 50 μm. **(B)** Quantitative analysis indicating that GABA_A_Rα1 levels in GCs are significantly lower in the DGs of APP^-/-^ mice than in those of WT controls (mean ± SEM; n=3 mice of WT controls and n=4 mice of APP^-/-^ mice; Student’s t-test, P<0.001). ***P < 0.001. **(C)** Representative confocal images of GCs in the DG of WT (black) and APP^-/-^ (red) mice showing alpha-amino-3-hydroxy-5-methylisoxazole-4-propionate receptor (AMPAR1) in red and gamma-aminobutyric acid A receptor alpha 5 (GABA_A_Rα5) in green and DAPI (chromatin) in blue. Scale bar: 50 μm. **(D, E)** Quantitative analysis indicating that GABA_A_Rα5 levels in GCs are significantly lower in the DGs of APP^-/-^ mice than in those of WT controls (means ± SEM; *n*=5 mice of both WT controls and APP^-/-^ mice; Student’s *t-*test, *P*<0.01); the fluorescence intensity (FI) of AMPAR1 was unchanged (mean ± SEM; *n*=7 mice of both WT controls and APP^-/-^ mice; Student’s *t*-test, *P*=0.664). APP: Amyloid precursor protein; DAPI: 4′,6-diamidino-2-phenylindole; DG: dentate gyrus; WT: wild-type.

***Additional Figure 2:*** *BDNF and p-TrkB levels in the DGs of WT and App*^*–/–*^ *mice.*

Additional Figure 2 BDNF and p-TrkB levels in the DGs of WT and APP^-/-^ mice.
(A) Representative confocal images of GCs in the DG of WT (black) and APP^-/-^ (red) mice showing BDNF in green and TrkB in red and DAPI (chromatin) in blue. Scale bar: 20 μm. **(B, C)** Quantitative analysis indicating that TrkB levels in GCs are significantly increased in the DGs of APP^-/-^ mice than in those of WT controls (mean ± SEM; *n*=6 mice of WT controls and *n*=5 mice of APP^-/-^ mice; Student’s *t*-test, *P*<0.01), **P<0.01; the fluorescence intensity (FI) of BDNF was unchanged (mean ± SEM; *n*=6 mice of WT controls and n=5 mice of APP^-/-^ mice; Student’s *t*-test, *P*=0.857). APP: Amyloid precursor protein; BDNF: brain-derived neurotrophic factor; DAPI: 4′,6-diamidino-2-phenylindole; DG: dentate gyrus; GCs: granule cells; TrkB: tropomyosin-related kinase receptor type B; WT: wild-type.

## Data Availability

*All relevant data are within the paper and its Additional files.*
